# Conditional deletion of Stat3 in mammary epithelium impairs the acute phase response and modulates immune cell numbers during post-lactational regression

**DOI:** 10.1002/path.3961

**Published:** 2012-01-27

**Authors:** Katherine Hughes, Julie A Wickenden, Judith E Allen, Christine J Watson

**Affiliations:** 1 Department of Pathology, University of CambridgeUK; 2 Centre for Immunity, Infection and Evolution, and Institute for Immunology and Infection Research, School of Biological Sciences, University of EdinburghUK

**Keywords:** mammary gland, involution, Stat3, inflammation, macrophage, signalling, acute-phase response, breast cancer

## Abstract

Mammary gland regression following weaning (involution) is associated with extensive cell death and the acquisition of an inflammatory signature. Characterizing the interplay between mammary epithelial cells, the re-emerging stroma and immune cells has implications for the understanding of the pathogenesis of pregnancy-associated breast cancer. *Stat3* has a role in orchestrating cell death and involution, and we sought to determine whether expression of Stat3 by the mammary epithelium also influences the innate immune environment and inflammatory cell influx in the gland. We examined mice in which *Stat3* is conditionally deleted only in the mammary epithelium. Distinct sets of genes associated with the acute phase response and innate immunity are markedly up-regulated during first phase involution in a Stat3-dependent manner. During second phase involution, chitinase 3-like 1, which has been associated with wound healing and chronic inflammatory conditions, is dramatically up-regulated by Stat3. Also at this time, the number of mammary macrophages and mast cells increases per unit area, and this increase is impaired in the absence of epithelial Stat3. Furthermore, expression of arginase-1 and Ym1, markers of alternatively activated macrophages, is significantly decreased in the absence of Stat3, whilst iNOS, a marker associated with classically activated macrophages, shows significantly increased expression in the Stat3-deleted glands. Thus, Stat3 is a key transcriptional regulator of genes associated with innate immunity and wound healing and influences mammary macrophage and mast cell numbers. The presence of epithelial Stat3 appears to polarize the macrophages and epithelial cells towards an alternatively activated phenotype, since in the absence of Stat3, the gland retains a phenotype associated with classically activated macrophages. These findings have relevance to the study of pregnancy-associated breast cancer and the role of Stat3 signalling in recruitment of alternatively activated tumour-associated macrophages in breast cancer. Copyright © 2012 Pathological Society of Great Britain and Ireland. Published by John Wiley & Sons, Ltd.

## Introduction

Mammary gland involution comprises the retrograde change of the organ to its pre-pregnant state following weaning. Involution is precisely orchestrated and controlled by a number of soluble factors, such as IL-6 cytokine family members leukaemia inhibitory factor (LIF) 1 and oncostatin M (OSM) 2, together with transforming growth factor-β3 3. Transcription factors involved in the control of involution include signal transducer and activator of transcription 3 (Stat3) 4–6, CCAAT/enhancer binding protein-δ 7 and nuclear factor-κB (NF-κB) 8.

Mammary gland involution has reversible and irreversible phases. During the first phase, milk stasis and local factors initiate extensive cell death but the dam retains the potential to recommence lactation. A subsequent transition to irreversible involution involves a second wave of cell death, coupled with dramatic glandular remodelling 9.

Stat3 has a pivotal role in mediating involution and mice with a mammary-specific conditional deletion of Stat3 exhibit a notable delay in this process 4, 5. Although mammary Stat3 is expressed throughout a reproductive cycle, it is specifically activated by tyrosine phosphorylation transiently on the day of birth and 6–12 h after weaning. The initial activator of Stat3 in the mammary gland is LIF 1, 10 and during reversible involution Stat3 regulates lysosomal-mediated programmed cell death in the mammary epithelial cells 6. During the irreversible phase, Stat3 is activated by OSM and its receptor (OSMR). OSMR itself is regulated by LIF-induced Stat3, in a positive feedback loop which ensures continued activation of Stat3 as LIF levels decline 2, 4.

At the onset of involution there is a dramatic up-regulation of genes associated with the acute phase response and inflammation 11, 13. As a sub-group of these genes have a microarray expression profile which mirrors that of Stat3 activation by phosphorylation 11, 12, it has been suggested, but not demonstrated, that these genes are Stat3 targets 11.

Macrophages have been highlighted as key players in the postnatal development of the mammary gland 14–17, and in remodelling associated with involution 13, 18, 19, 20. Macrophage infiltration occurs from day 3 of involution 13, 18, 19 and investigators have described an eight-fold increase in macrophage number 20. In parallel to studies emphasizing the role of macrophages in second phase involution, other authors have described the acquisition of certain inflammatory cell characteristics by the mammary epithelial cells themselves, particularly expression of the lipopolysaccharide receptor CD14 13, which may enable epithelial cells to adopt a phagocytic phenotype during involution 13, 21.

Mast cells also have a role in mammary gland development in both puberty 22 and involution 23. Mast cell granules contain plasma kallikrein, considered the dominant plasminogen activator during mammary involution 23. Mice in which plasma kallikrein activity has been inhibited exhibit a delay in involution, manifested by retarded epithelial cell death and delayed re-emergence of adipocytes 23.

Given the up-regulation of acute phase response genes in first phase involution and the increase in inflammatory cell numbers seen in second phase involution, the process of mammary gland regression post-weaning has been likened to a wound-healing response 13. There is a well-established association between inflammation and neoplastic transformation in many organ systems 24, and it has been recently demonstrated in a mouse model that the inflammatory environment of the mammary gland during involution has pro-tumourigenic potential 25–28. Human epidemiological evidence demonstrates that for the first 3–5 years post partum, there is a transient increase in risk of breast cancer 29–31, with this risk increasing for women who delay their first pregnancy until 35 years of age or more 32. The clinical term ‘pregnancy-associated breast cancer’ (PABC), is variably defined as mammary neoplasia which is diagnosed during pregnancy or up to 1–5 years post partum 30, 33, 34. A subset of cases of PABC arise in the immediate post-partum period, and these patients are considered to have the worst prognosis 35.

Understanding the tissue remodelling and inflammatory changes which occur during involution is clearly crucial to the understanding of the pathogenesis of PABC. Given the pivotal role for Stat3 in orchestrating mammary regression post-weaning, we hypothesized that mammary epithelial expression of Stat3 will influence the gland's inflammatory signature. To test this assertion, we have used mice with a conditional mammary epithelial deletion of Stat3.

## Materials and methods

### Animal husbandry

Mice carrying the *Stat3* gene flanked by loxP sites 36 were crossed with a strain containing a β-lactoglobulin promoter-driven *Cre* gene 37, as previously described 6. Mouse husbandry, induction of involution and tissue collection were as previously described 6. At least three mice were used for each time point in every experiment, except in Figure 6C (two mice/time point). All animals were treated according to local ethical committee and UK Home Office guidelines. Tissues from mice with deletion of the OSM receptor (*OSMR*^−/−^) 2 were a generous gift from Dr Richard Clarkson, School of Biosciences, University of Cardiff, UK.

### Microarray analysis

Previously published Affymetrix microarray data was used to compare mammary tissue from 12 developmental stages 12.

### Histology

Tissue for sectioning was harvested from the abdominal glands, and was fixed for 24 h in 4% formaldehyde in PBS (Sigma), sectioned and stained with haematoxylin and eosin (H&E) or toluidine blue, following standard protocols.

### Immunofluorescence

Immunofluorescence was carried out on sections of paraffin-embedded tissue, as previously described 38. The following primary antibodies were used: anti-CD14 (AbCam 25 092); anti-E-cadherin (BD Biosciences 610 182); anti-liver arginase 1 (AbCam 91 279); anti-iNOS (Thermo-Fisher Scientific PA1-036); and anti-Ym1 39.

### Immunohistochemistry

Paraffin-embedded sections were deparaffinized in xylene, rehydrated in decreasing concentrations of ethanol and washed in distilled water, phosphate-buffered saline (PBS), 0.5% Triton PBS, and PBS. Endogenous peroxidase activity was quenched by incubation in 0.3% hydrogen peroxide (Sigma). Trypsin antigen retrieval was employed (AbCam). Sections were blocked sequentially in 5% rabbit serum and avidin D and biotin block (both from Vector Laboratories). Sections were incubated overnight at 4 °C with rat anti-F4/80 (AbCam 6640) or negative control rat IgG, both in 2% rabbit serum in PBS. Biotin-conjugated polyclonal rabbit anti-rat IgG secondary antibody was used (AbCam 6733-1). The avidin and biotinylated horseradish peroxidase macromolecular complex technique (Vectastain ABC Kit; Vector Laboratories) with peroxidase substrate (Zymed Laboratories, Invitrogen) was used for immunohistochemical visualization and the sections were counterstained with haematoxylin.

### Quantitative RT–PCR (qRT–PCR)

RNA extraction, complementary DNA synthesis and qRT–PCR were carried out as previously described 40. Expression levels of selected genes were measured. Primer sequences are detailed in Table S1 (see Supporting information).

### Immunoblotting

Sample preparation and immunoblotting were carried out as previously described 41. The following antibodies were used: anti-phospho-Stat3 (Tyr705; Cell Signalling Technologies 9131); anti-Stat3 (BD Transduction Laboratories, 610 190); anti-chitinase 3-like 1 (R&D Systems, MAB2649); anti-liver arginase 1 (AbCam 91 279); and anti-β-actin (AbCam 8227).

### Cell culture

EpH4 cells were grown in Dulbecco's modified Eagle's medium (DMEM; Gibco) containing 10% fetal calf serum (FCS; Sigma). At 50% confluence, the cells were stimulated with a final concentration of 25 ng/ml recombinant mouse oncostatin M (495-MO; R&D Systems) or carrier (0.0001% BSA in PBS), and protein was prepared from cell lysates.

## Results

### Stat3 regulates expression of genes associated with the acute phase response in first phase involution

**Figure 1:**
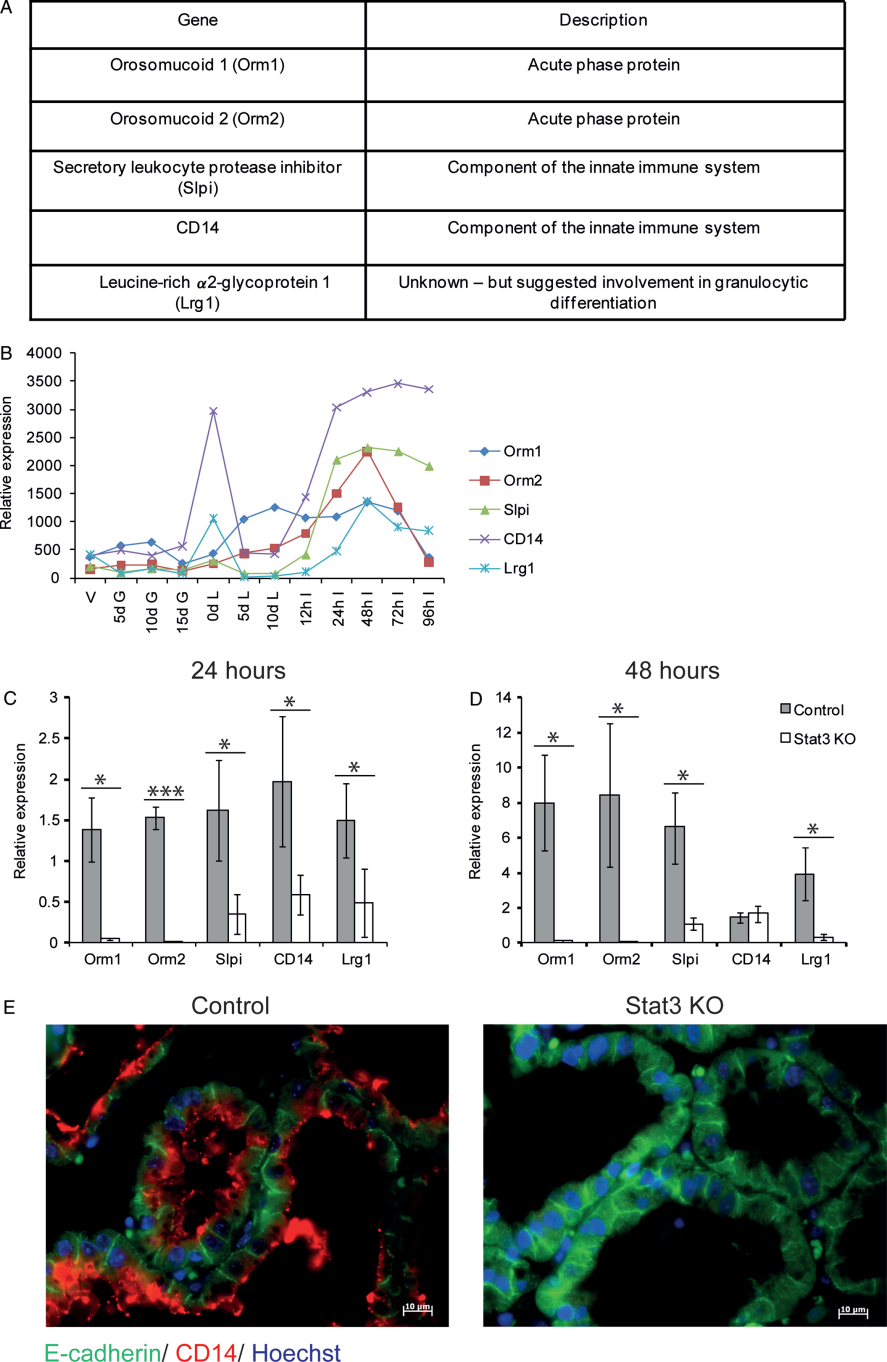
Acute phase response genes are up-regulated in first phase involution and a subset of these genes are Stat3-regulated. Table (A) and microarray expression data (B) detailing genes associated with innate immunity and the acute phase response which show a dramatic increase in expression in the first phase of involution. Microarray profiles were derived from 12 different time points in the developmental cycle of the mouse mammary gland; V, virgin; dG, days of gestation; dL, days of lactation; hI, hours of involution. Expression of selected genes associated with the acute phase response and innate immunity at 24 h (C) and 48 h (D) of involution in control and Stat3 KO mice, measured by qRT–PCR relative to expression of cyclophilin (a housekeeping gene); values are mean ± standard deviation (SD) from at least three biological repeats; *p < 0.05, ***p < 0.001, as determined by Student's *t*-test. (E) Immunofluorescence staining for CD14 (red), E-cadherin (green) and DNA (Hoechst; blue) on control and Stat3 KO tissue at 24 h of involution; scale bars = 10 µm

Previously published Affymetrix microarray data 12 indicate that there is a dramatic up-regulation of numerous genes associated with the acute phase response and inflammation at the onset of involution. These include acute phase response genes orosomucoids 1 and 2 (Orm1 and 2), the immunomodulatory secretory leukocyte protease inhibitor (Slpi), CD14 and leucine-rich α2-glycoprotein 1 (Lrg1) 42 (Figure 1A, B).

In order to determine whether Stat3 activity influenced the expression of genes associated with the acute phase response, we compared mice with a mammary-specific conditional deletion of Stat3 (Stat3^fl/fl^; BLG–Cre, hereafter called Stat3 KO) to mice with the Stat3 allele flanked by loxP sites but with no expression of Cre (Stat3^fl/fl^, hereafter called control). Using qRT–PCR, we analysed expression of the panel of acute phase response genes from mammary tissue. At 24 and 48 h of involution, expression of Orm1 and 2, Slpi and Lrg1 is significantly reduced in the Stat3 KO tissue (Figure 1C, D). This finding is notable, as it suggests that Stat3, expressed by the mammary epithelial cells themselves, is responsible for the acute phase response and regulation of the inflammatory environment of the gland during early involution.

CD14 is expressed at the apical surface of the mammary luminal epithelial cells from 24 h and throughout the first 4 days of involution, as previously described 13. However, this expression is not detected in the absence of Stat3 (Figure 1E; see also Supporting information, Figure S1A–D). Although CD14 expression is most commonly associated with macrophages and dendritic cells, previous investigators have suggested that luminal mammary epithelial CD14 expression during involution indicates acquisition of a phagocytic phenotype 13, which may facilitate epithelial cell clearance of shed cells from the alveolar lumina 21. Our data suggest that the up-regulation of CD14 expression is an essential component of the Stat3 cell death involution programme, and that epithelial cells lacking intact Stat3 signalling pathways may have an impaired capacity for efferocytosis.

Interestingly, although at 24 h of involution expression of *CD14* mRNA is significantly reduced in the Stat3 KO tissue (Figure 1C), this is not the case at 48 h (Figure 1D) or 96 h (authors' unpublished data), in spite of the strong association between Stat3 activity and CD14 protein expression observed by immunofluorescence (see Supporting information, Figure S1B–D). This suggests that post-transcriptional mechanisms are likely to be important in the regulation of CD14 expression by Stat3.

### Stat3 regulates expression of chitinase 3-like 1 (*Chi3L1*), a gene associated with wound healing, in second phase involution

As we observed dramatic regulation of expression of acute phase response genes by Stat3 in the first phase of involution, we wished to explore the potential Stat3 target genes associated with the chronic inflammatory ‘wound-healing signature’ in its second phase. Chi3L1 (synonym: BRP-39) and the human homologue YKL-40, is a protein originally identified in mouse breast cancer cells, which has been associated with a range of chronic inflammatory and allergic conditions and wound healing 43. Expression has been associated with macrophages, neutrophils and fibroblasts 43. Since Chi3L1 expression was suggested to be up-regulated during involution by microarray analysis 13, we wished to investigate a potential connection with Stat3 activity.

**Figure 2:**
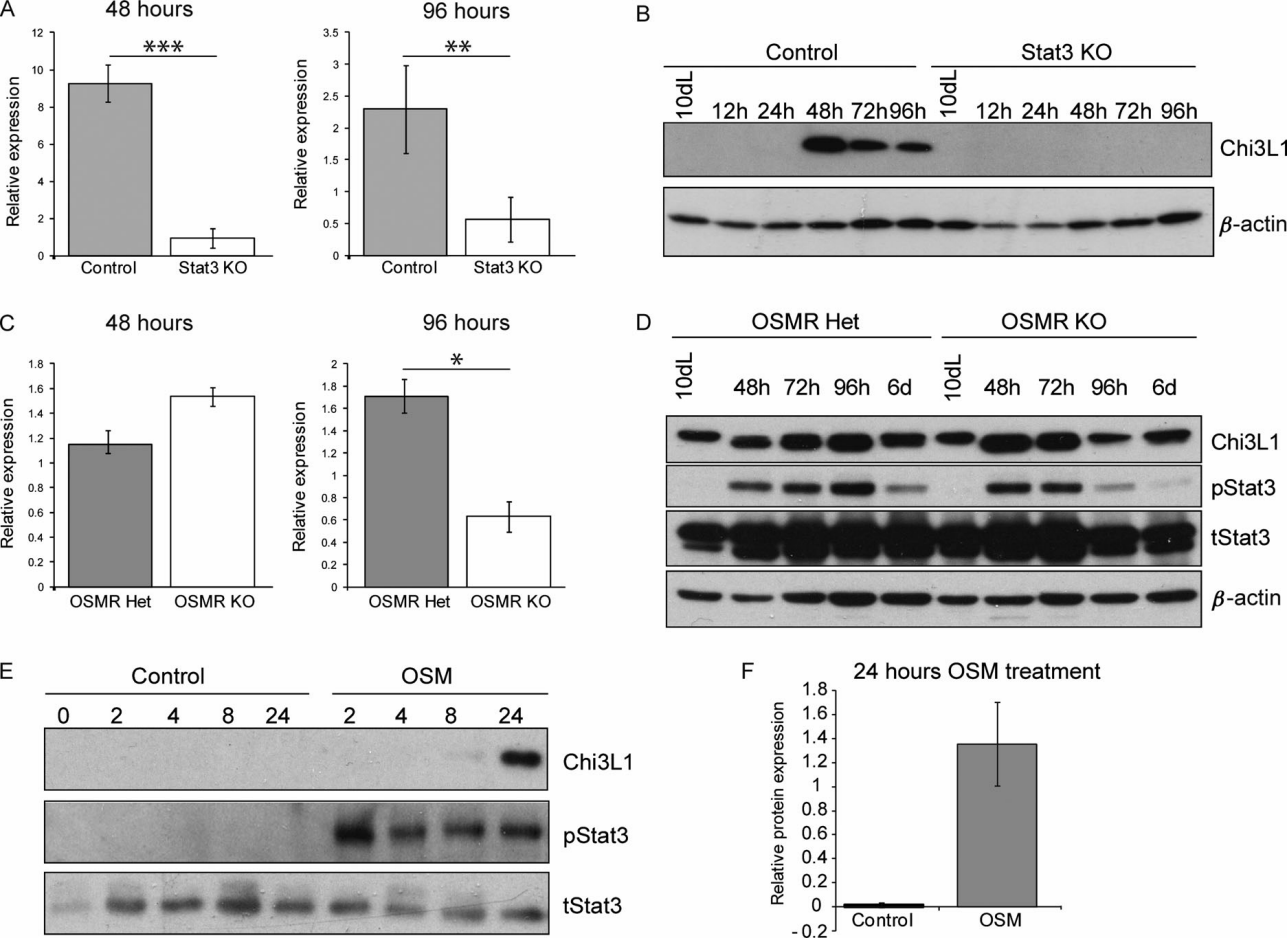
Chitinase 3-like 1 (Chi3L1) is up-regulated at the onset of the second phase of involution in a Stat3-dependent manner. (A) Expression of Chi3L1 in control and Stat3 KO mice at 48 and 96 h of involution measured by qRT–PCR relative to expression of cyclophilin (a housekeeping gene); values are mean ± SD from at least four biological repeats; **p < 0.01, ***p < 0.001, as determined by Student's *t*-test. (B) Representative western blot analysis, showing Chi3L1 and β-actin expression at 10 days of lactation (10 dL) and during involution with control and Stat3 KO tissues; h, hours of involution. (C) Expression of Chi3L1 in control (OSMR Het) and OSMR KO mice at 48 and 96 h of involution, measured by qRT–PCR relative to expression of cyclophilin (a housekeeping gene); values are mean ± SD from at least three biological repeats; *p < 0.05, as determined by Student's *t*-test. (D) Representative western blot analysis, showing expression of Chi3L1, phosphorylated Stat3 (pStat3), total Stat3 (tStat3) and β-actin at 10 dL and in involution with control (OSMR Het) and OSMR KO tissues; h, hours of involution; d, days of involution. (E) Western blot analysis of mammary epithelial EpH4 cells, stimulated with oncostatin M (OSM) or vehicle alone (control) for the indicated number of hours; blot is representative of two experimental repeats. (F) Quantification of two western blots illustrating Chi3L1 expression relative to tStat3, following 24 h of treatment of EpH4 cells with OSM or vehicle alone (control)

Expression of *Chi3L1* mRNA is significantly re- duced in Stat3 KO animals at 48 and 96 h of involution (Figure 2A). Chi3L1 protein expression is observed from 48 h of involution in control mice but expression is undetectable in the Stat3 KO animals (Figure 2B). This is interesting, as 48 h marks the switch point to irreversible involution in mice 44 and is the point at which the phenotype of the gland becomes increasingly similar to a healing wound.

As the cytokine oncostatin M (OSM) is the principal activator of Stat3 in second phase involution, we investigated expression of Chi3L1 in mice with total deletion of the OSM receptor (OSMR) 2 (Figure 2C, D). At 48 h of involution, levels of *Chi3L1* mRNA and protein do not differ between control mice and those with deletion of OSMR (OSMR KO) (Figure 2C, D), which is consistent with the observation that the OSMR KO mice have unperturbed Stat3 activity for the first 2 days of involution 2, when LIF signalling is the predominant activator of Stat3 10. However, at 96 h of involution, mRNA and protein expression of *Chi3L1* is notably reduced in OSMR KO mice (Figure 2C, D), a profile consistent with the abrogation of Stat3 activity in the OSMR KO mice as LIF levels decline in second phase involution 10 and OSM becomes the principal activator of Stat3 2. Additionally, mammary epithelial EpH4 cells stimulated with OSM for 24 h to activate Stat3 exhibit increased expression of Chi3L1 compared to those cells stimulated with vehicle alone (Figure 2E, F).

Taken together, these results suggest that Chi3L1 is downstream of Stat3 signalling induced by OSM in second-phase involution. However, by 6 days of involution, levels of Chi3L1 are comparable between control and OSMR KO animals, although pStat3 expression is negligible in the latter (Figure 2D). This is interesting, as it suggests that Stat3 is not the sole regulator of Chi3L1 expression in mammary gland involution. Further evidence of this is the finding that Stat3 KO mice will express Chi3L1 if pups are returned to the dam following 48 h of involution (authors' unpublished data). Given the established role of NF-κB in regulation of involution 8, and as this transcription factor has been suggested to regulate secretion of Chi3L1 in chondrocytes 45, we hypothesize that NF-κB may influence Chi3L1 expression in later involution.

### In the absence of epithelial Stat3 during involution, the number of macrophages and mast cells is diminished

Having observed that Stat3 has a pivotal role in control of the inflammatory environment of the gland during involution, we wished to consider whether the diminished inflammatory gene expression signature in Stat3 KO mice influenced the number of immune cells present. In order to investigate the fluctuations in macrophage and mast cell numbers within the gland, we performed immunohistochemical staining for F4/80 and histochemical staining with toluidine blue for macrophages and mast cells, respectively.

**Figure 3:**
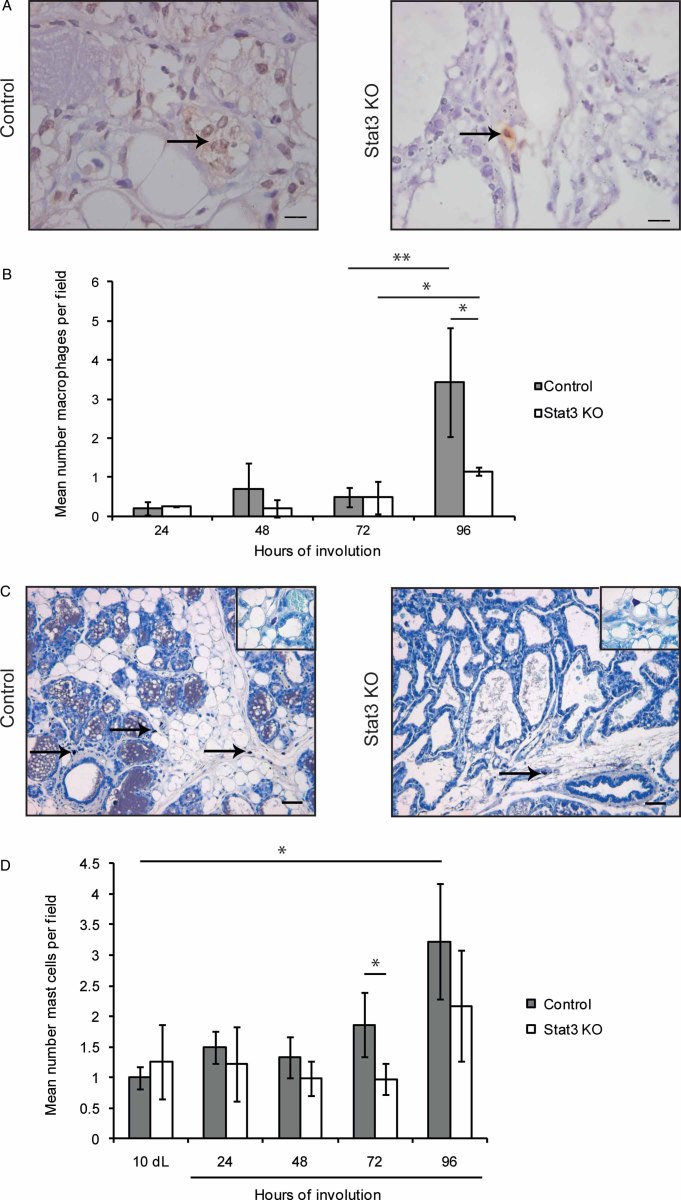
In the absence of epithelial Stat3, the increase in numbers of macrophages and mast cells during involution is diminished. (A) Immunohistochemical staining for F4/80 to identify murine macrophages (arrows) in control and Stat3 KO glands at 96 h of involution; scale bars = 10 µm. (B) Quantification of macrophages per × 1000 field. A minimum of 20 fields and three mice were analysed per time point; values are mean ± SD from at least three biological repeats; *p < 0.05, **p < 0.01, as determined by Student's *t*-test. (C) Toluidine blue staining for mast cells (arrows) in control and Stat3 KO glands at 72 h of involution; scale bars = 50 µm; inset shows mast cells at × 1000 magnification. (D) Quantification of mast cells per × 400 field; a minimum of 20 fields and 3 mice were analysed per time point; values are mean ± SD from at least three biological repeats; *p < 0.05, as determined by Student's *t*-test

There are significantly more macrophages per unit area in control mice at 96 h compared to 72 h involution, with a more than six-fold increase in numbers between these time points (Figure 3A, B). This is in accordance with previously published data, allowing for strain variations between rodent models 13, 18, 19, 20. The increase in macrophage numbers is seen during the second phase of involution, when the gland becomes irreversibly committed to a profound degree of matrix remodelling and cell death. It is likely that the role of macrophages encompasses both stromal reorganization and phagocytosis of cellular remnants. The increase in macrophage numbers per unit area is more modest in the Stat3 KO glands, and at 96 h control glands have significantly more macrophages than those lacking epithelial Stat3 (Figure 3A, B).

Mast cells are also present in the gland throughout involution and there are increased numbers of mast cells at 96 h of involution compared to 10 days lactation in control glands. However, in Stat3 KO glands there are significantly reduced numbers of mast cells at 72 h of involution (Figure 3C, D).

The decrease in numbers of macrophages and mast cells in Stat3 KO glands compared to controls at 96 h and 72 h involution, respectively, is interesting as it suggests that epithelial Stat3 is either directly or indirectly influencing the numbers of these immune cells in the gland during the irreversible phase of involution. This may be a consequence of reduced expression of cytokines and/or chemokines by the epithelium, or lack of appropriate feed-forward mechanisms from resident tissue macrophages and mast cells within the mammary gland, due to reduced numbers of dead epithelial cells.

### In the absence of epithelial Stat3, expression of matrix metalloproteinases 2, 3 and 9 is significantly reduced

**Figure 4:**
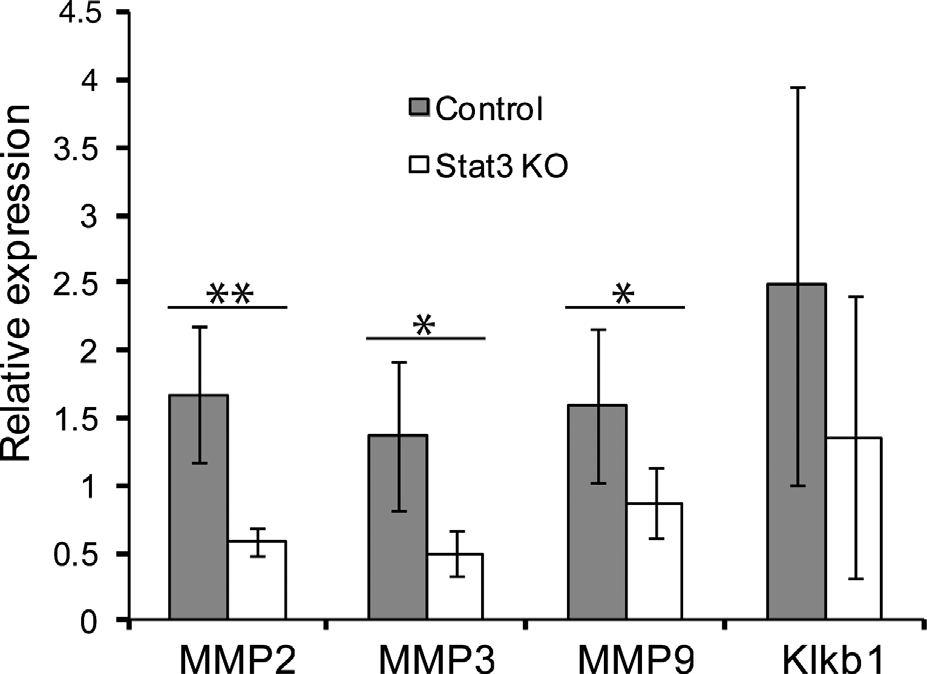
At 96 h of involution, expression of matrix metalloproteinase enzymes 2, 3 and 9 is down-regulated in the absence of epithelial Stat3. Expression of matrix metalloproteinases 2, 3 and 9 (MMP2, MMP3 and MMP9) and prekallikrein (Klkb1) in control and Stat3 KO mice at 96 h of involution measured by qRT–PCR relative to expression of cyclophilin (a housekeeping gene); values are mean ± SD from five biological repeats; *p < 0.05, **p < 0.01, as determined by Student's *t*-test

Macrophages have previously been demonstrated to reshape the stromal architecture via elaboration of matrix metalloproteinases (MMPs) 18, and we therefore considered it likely that a reduction in expression of matrix metalloproteinases from diminished numbers of macrophages would contribute to the delayed involution phenotype seen in Stat3 KO glands. In order to test this hypothesis, we used qRT–PCR to measure expression of MMPs 2, 3 and 9 at 96 h of involution, and demonstrated a significant reduction in expression of these enzymes in the Stat3 KO glands (Figure 4). Our results are in accordance with an earlier study demonstrating reduced expression of MMP3 in OSMR KO mice 2.

Given that plasminogen-deficient mice exhibit de- layed involution 46, and that the predominant mammary plasminogen activator during involution (plasma kallikrein) localizes to mammary mast cells 23, we wished to investigate whether the reduction in mast cell numbers observed in the Stat3 KO mice has an effect on expression of the prekallikrein gene (Klkb1). Although Klkb1 expression measured at 96 h of involution by qRT–PCR does not differ significantly between control and Stat3 KO glands, there is a trend towards reduced expression in the Stat3 KO animals (Figure 4). Interestingly, we observe considerable mouse-to-mouse variation in expression of Klkb1.

### iNOS expression is increased in second phase involution in the absence of epithelial Stat3 activity

**Figure 5:**
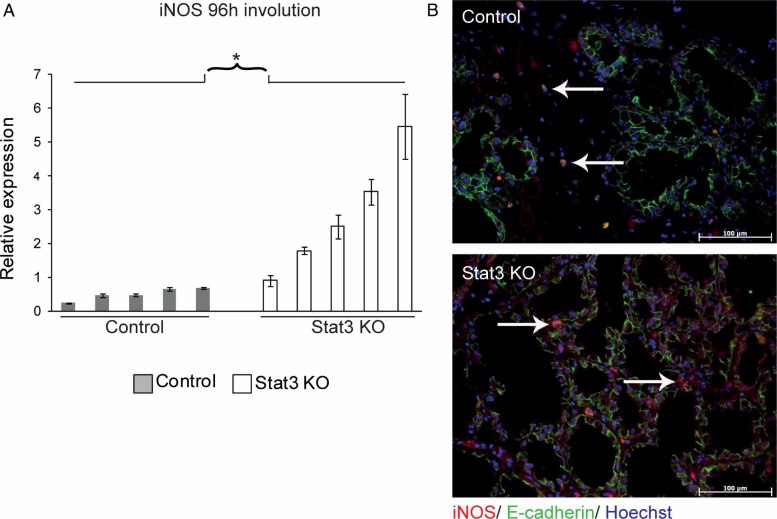
Absence of epithelial Stat3 activity increases iNOS expression during involution. (A) Expression of inducible nitric oxide synthetase (iNOS) at 96 h of involution in control and Stat3 KO mice, measured by qRT–PCR relative to expression of cyclophilin (a housekeeping gene); values are mean ± SD from at least three experimental repeats, with each bar representing an individual mouse; *p < 0.05, as determined by Student's *t*-test. (B) Immunofluorescence staining for iNOS (red), E-cadherin (green) and DNA (Hoechst; blue) at 96 h of involution on control and Stat3 KO tissue. Arrows indicate cytoplasmic iNOS expression within cells with morphology consistent with macrophages; scale bars = 100 µm

In order to investigate the phenotype of the involution macrophages, we used qRT–PCR and immunofluorescence to analyse expression of iNOS [associated with a classically activated (M1) macrophage phenotype] 47. At 10 days of lactation, mRNA levels of *iNOS* are not significantly different between control and Stat3 KO glands (see Supporting information, Figure S5). At 96 h of involution, mRNA levels for iNOS are significantly elevated in the Stat3 KO tissue (Figure 5A), suggesting that absence of epithelial Stat3 favours polarization towards an M1 phenotype. In both control and Stat3 KO glands, iNOS expression predominantly localizes to the cytoplasm of cells, with location and morphology consistent with macrophages (Figure 5B; see also Supporting information, Figure S2).

### Epithelial Stat3 expression influences polarization to an alternatively activated macrophage phenotype during involution

**Figure 6:**
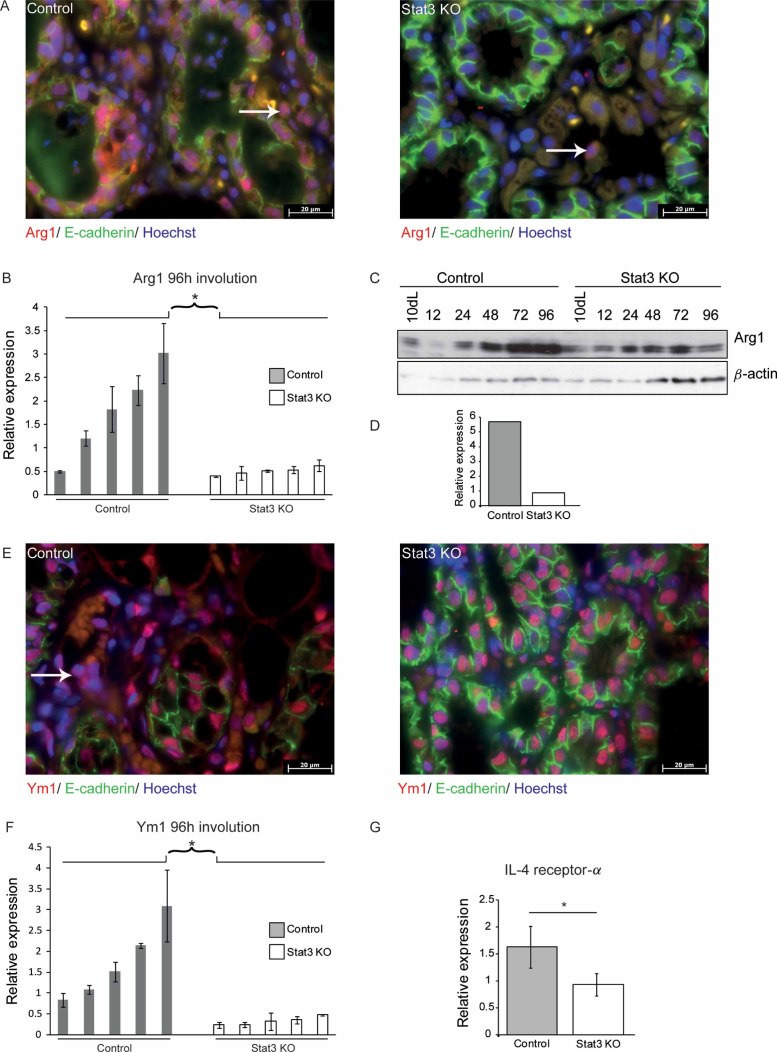
Epithelial Stat3 activity influences polarization to an alternatively activated macrophage phenotype during involution. (A) Immunofluorescence staining for arginase-1 (red), E-cadherin (green) and DNA (Hoechst; blue) at 96 h of involution on control and Stat3 KO tissue. Arrows indicate arginase-1 expression within cells with morphology consistent with macrophages; scale bars = 20 µm. (B) Expression of arginase-1 (Arg1) at 96 h of involution in control and Stat3 KO mice, measured by qRT–PCR relative to expression of cyclophilin (a housekeeping gene); values are mean ± SD from at least three experimental repeats, with each bar representing an individual mouse; *p < 0.05, as determined by Student's *t*-test. (C) Representative western blot analysis of two independent biological samples, showing arginase-1 (Arg1) and β-actin expression at 10 days of lactation (10 dL) and in involution with control and Stat3 KO tissues (values indicate hours of involution). (D) Quantification of western blot from Figure 6C, illustrating Arg1 expression relative to β-actin at 96 h of involution. (E) Immunofluorescence staining for Ym1 (red), E-cadherin (green) and DNA (Hoechst; blue) at 96 h of involution on control and Stat3 KO tissue. Arrow indicates Ym1 expression within a cluster of cells adjacent to a blood vessel, which have morphology consistent with macrophages; scale bars = 20 µm. (F) Expression of Ym1 at 96 h of involution in control and Stat3 KO mice measured by qRT–PCR relative to expression of cyclophilin (a housekeeping gene); values are mean ± SD from at least three experimental repeats, with each bar representing an individual mouse; *p < 0.05, as determined by Student's *t*-test. (G) Expression of IL-4 receptor-α at 96 h of involution in control and Stat3 KO mice, measured by qRT–PCR relative to expression of cyclophilin (a housekeeping gene); values are mean ± SD from five biological repeats; *p < 0.05, as determined by Student's *t*-test

As macrophages with an alternatively activated (M2) phenotype have been observed in involution 20, we wished to investigate whether Stat3 activity influenced this M2 polarization. To achieve this, we examined the expression of arginase-1 and Ym1 (associated with a murine macrophage M2 phenotype) 47. At 10 days of lactation, mRNA levels of both markers are similar between control and Stat3 KO glands (see Supporting information, Figure S5). However, at 96 h of involution, control tissue expresses significantly higher levels of arginase-1 than the Stat3 KO glands (Figure 6A–D). There are moderate levels of arginase-1 expression in macrophages and surprisingly high levels of arginase-1 expression in mammary epithelial cells. In Stat3 KO glands, arginase-1 is minimally detectable in the cytoplasm of macrophages and only occasional epithelial cells exhibit expression (Figure 6A; see also Supporting information, Figure S3). Similarly, at 96 h of involution, control tissue expresses significantly higher levels of Ym1 than the Stat3 KO glands (Figure 6E, F). Both mammary epithelial cells and macrophages express Ym1 in control glands and reduced levels of expression are observed in macrophages in Stat3 KO glands, although the level of epithelial expression remains high (Figure 6E; see also Supporting information, Figure S4).

The cytokines IL-4 and IL-13 have been implicated in the acquisition of an M2 phenotype 47 and an increase in levels of both IL-4 and IL-13 has been observed during involution in rats 20. Accordingly, we used qRT–PCR to investigate the expression of IL-4 receptor-α in control and Stat3 KO glands at 96 h of involution, and demonstrated a significant reduction in expression of IL-4 receptor-α in the Stat3 KO glands (Figure 6G).

Taken together, these results suggest that the absence of epithelial Stat3 modulates both macrophage and mammary epithelial cell acquisition of an M2 phenotype during involution. It is possible that this polarization is achieved through regulation of expression of IL-4 receptor-α, although this requires further experimental investigation.

## Discussion

Using mice with a mammary epithelial- and lactation-specific *Stat3* deletion, we have demonstrated that epithelial expression of Stat3 is critical for the induction of an inflammatory gene expression signature during involution. In the first phase of involution, Stat3 dramatically modulates expression of genes associated with the acute-phase response and innate immunity, and in the second phase, Stat3 regulates expression of chitinase 3-like 1, which is associated with chronic inflammation and wound healing 43.

It is interesting to note that during involution there are complex and overlapping roles for the epithelial and immune cell compartments. We have previously established that mammary epithelial cells undergo a transition from Th1 to Th2 cytokine production upon the induction of differentiation during development 41. Similarly, in the present study we demonstrate that epithelial Stat3 expression during involution drives a polarization of the epithelial and immune cell components of the gland towards an M2 phenotype.

Our results also give insights into mammary remodelling during involution. Other investigators have previously demonstrated mammary epithelial expression of CD14 and related this expression to acquisition of a phagocytic phenotype by the luminal epithelial cells 13. Here, we demonstrate the CD14 expression by mammary epithelial cells is critically dependent on Stat3 activity. As Stat3-dependent CD14 expression occurs as early as 24 h of involution, it would seem that epithelial efferocytosis may be an important mechanism for clearance of dead epithelial cells early in the first phase, when dying cells are shed into the alveolar lumen. At this stage, the shed cells are most likely inaccessible to macrophages, as the alveolar units appear largely intact by light microscopy. Later, as the number of macrophages within the gland increases, it seems likely that these professional phagocytes will clear cellular debris, particularly as the alveolar units collapse and structural integrity is lost, allowing macrophages unimpeded access to dying cells. As epithelial CD14 expression persists through the first 4 days of involution in control glands, we cannot discount an ancillary role for mammary epithelial cell-mediated efferocytosis during the irreversible phase.

In the absence of epithelial Stat3, reduced numbers of mast cells and macrophages are present at 72 and 96 h of involution, respectively. Given the fundamental role of Stat3 activity in up-regulating the early involution acute phase response, it is possible that immune cell recruitment is directly dependent on epithelial Stat3. However, it is also possible that the reduction in numbers of macrophages and mast cells in the absence of epithelial Stat3 may reflect an indirect effect of Stat3 signalling, viz a delay in restructuring the gland when Stat3 is conditionally deleted. The exact directionality of the relationship between Stat3 signalling, mammary remodelling and the increase in numbers of macrophages and mast cells is difficult to determine, particularly given that both epithelial cells 48 and macrophages 18 may elaborate matrix metalloproteinases responsible for stromal restructuring.

We have demonstrated that epithelial Stat3 activity has a direct or indirect effect upon both epithelial and macrophage expression of markers of an M2 macrophage phenotype during involution. This has important implications for the understanding of interactions between tumour-associated macrophages and neoplastic epithelial cells, particularly given that many breast tumours exhibit constitutive activation of Stat3 11. We hypothesize that Stat3 signalling within neoplastic breast epithelial cells themselves may be important for the recruitment of macrophages and polarization of these cells towards an M2 phenotype.

Finally, our results demonstrate that Stat3 signalling during both phases of the involution process is critical for the expression of an inflammatory signature within the gland. As it is this inflammatory signature which is considered to be the cause of the increased risk of occurrence of breast cancer in the period immediately post-weaning 25–28, we suggest that epithelial Stat3 expression is at the very heart of the pathogenesis of pregnancy-associated breast cancer.

## SUPPORTING INFORMATION ON THE INTERNET

The following supporting information may be found in the online version of this article:

**Figure S1.** Expression of CD14 is Stat3 regulated throughout the first 4 days of involution.

**Figure S2.** iNOS is expressed by macrophages during involution.

**Figure S3.** Epithelial Stat3 influences expression of arginase-1 during involution.

**Figure S4.** Epithelial Stat3 influences expression of Ym1 during involution

**Figure S5.** Expression of inducible nitric oxide synthase, arginase-1 and Ym1 does not vary between control and Stat3 KO mice at 10 days of lactation

**Table S1.** Primers used for quantitative RT–PCR analysis.
