# Supplementary figures and images for: Conditional deletion of Stat3 in mammary epithelium impairs the acute phase response and modulates immune cell numbers during post-lactational regression
Source: J Pathol. 2012 Jan 27;227(1):106–17. doi: 10.1002/path.3961 (PMC3477635; doi:10.1002/path.3961)

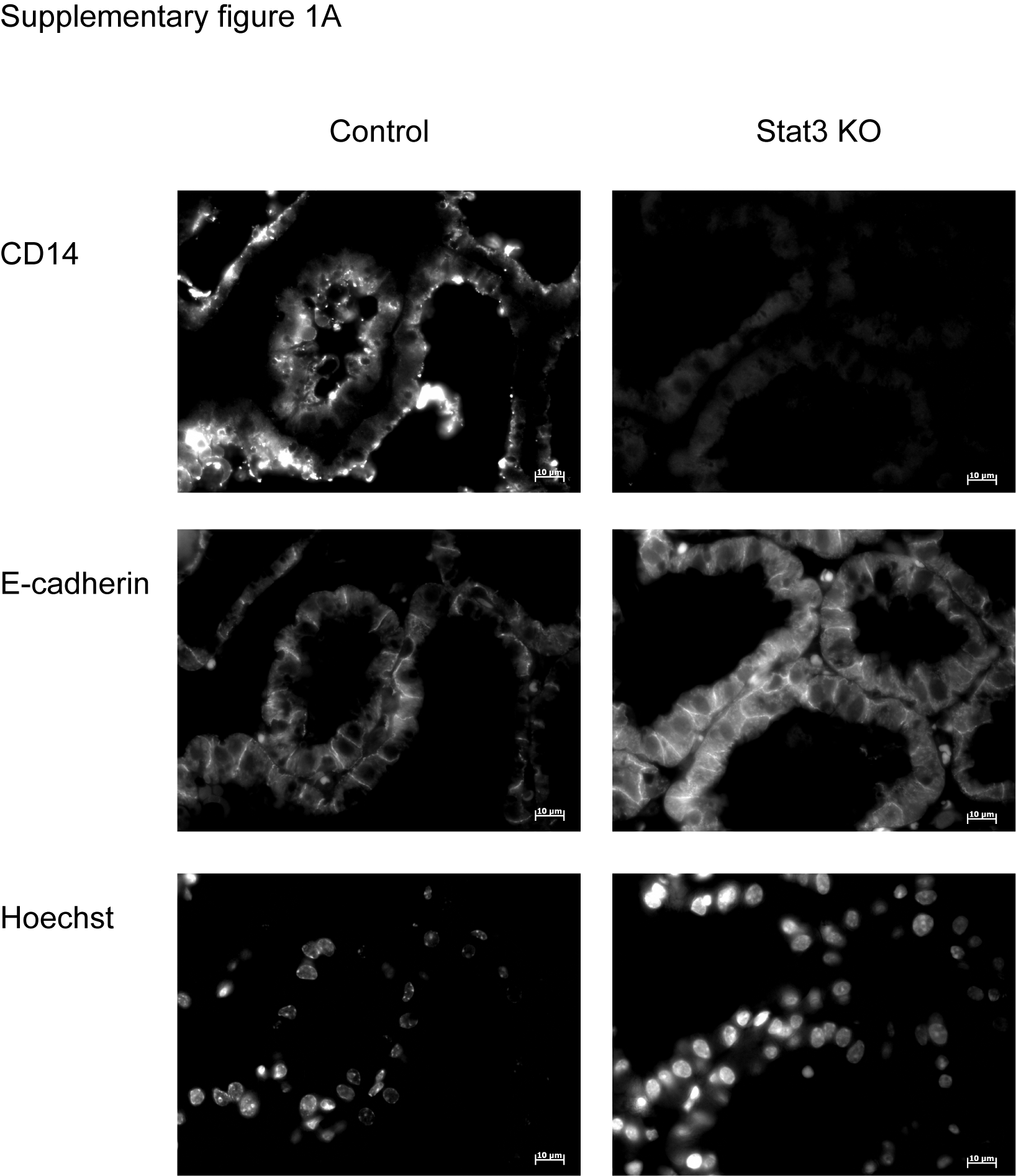

Supplement: Supplementary file 1 [file path0227-0106-SD1a.tif]

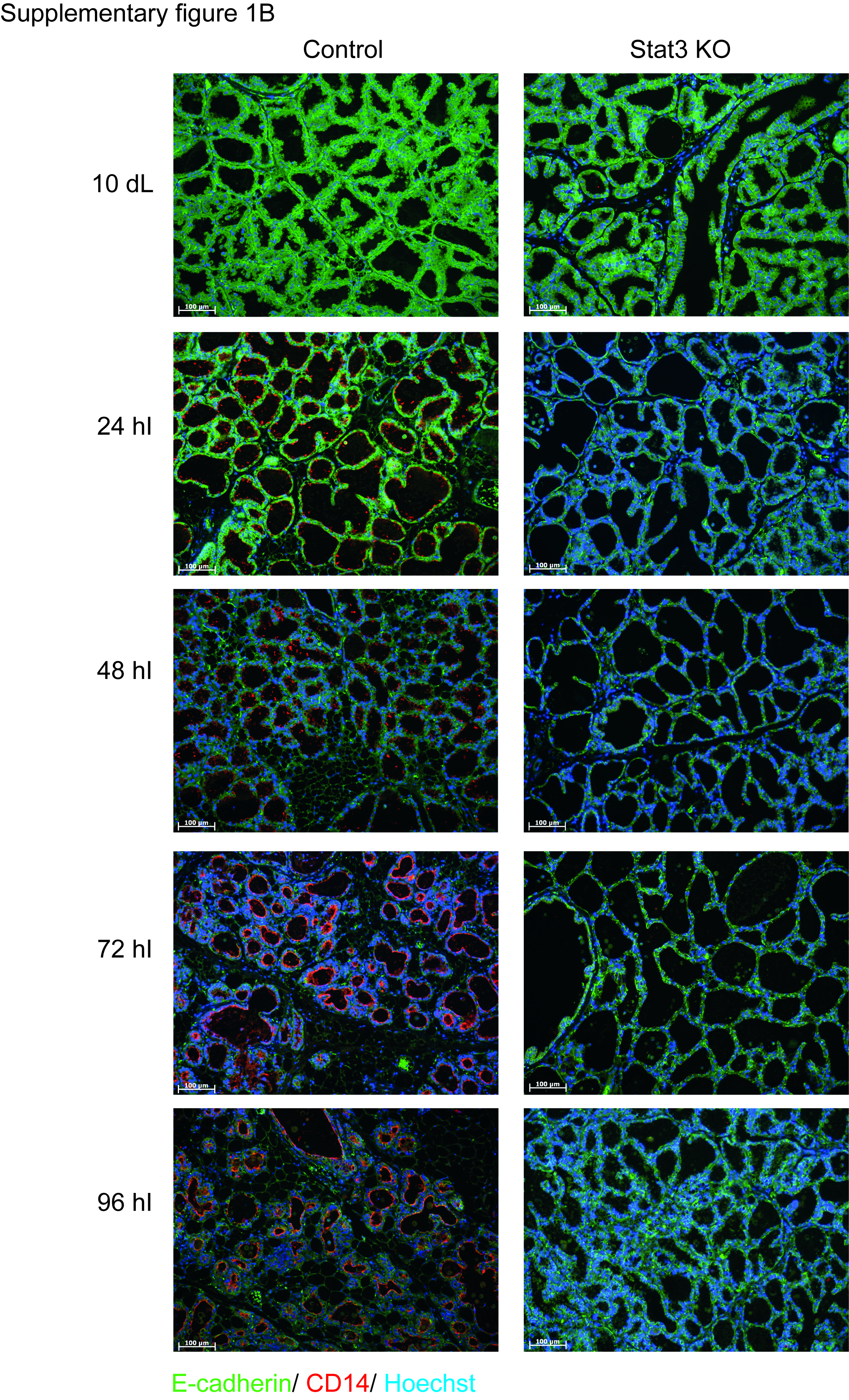

Supplement: Supplementary file 2 [file path0227-0106-SD1b.tif]

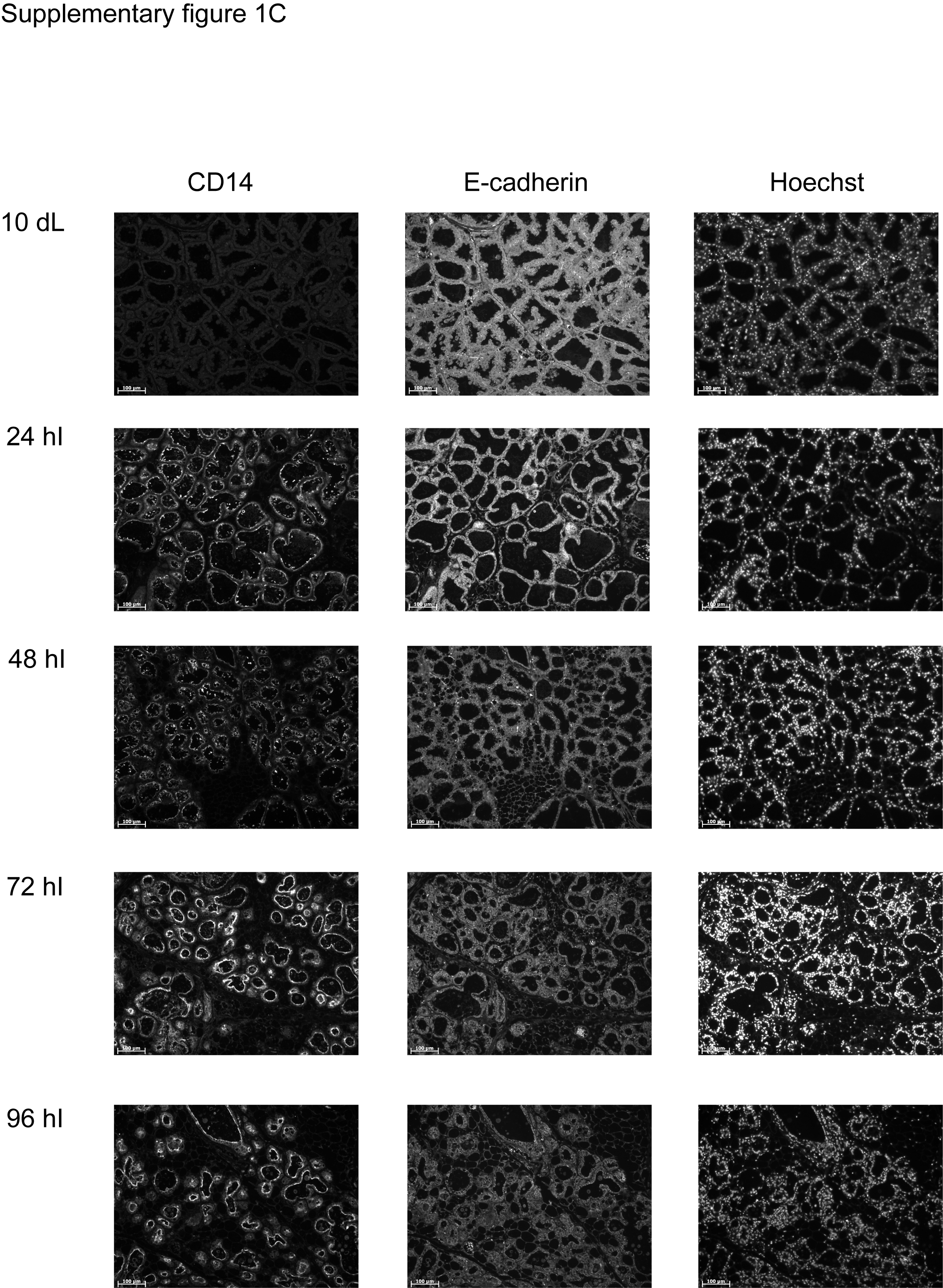

Supplement: Supplementary file 3 [file path0227-0106-SD1c.tif]

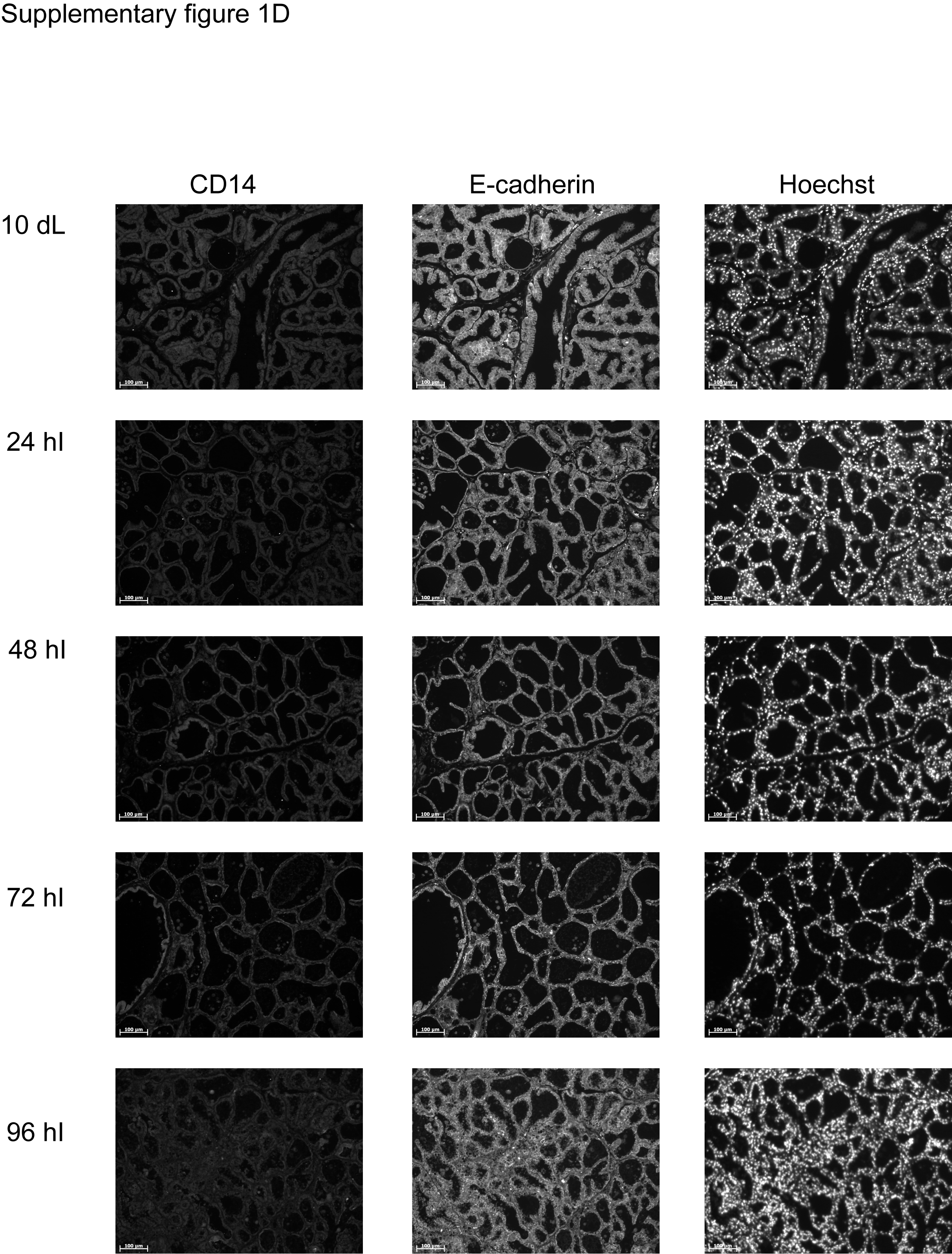

Supplement: Supplementary file 4 [file path0227-0106-SD1d.tif]

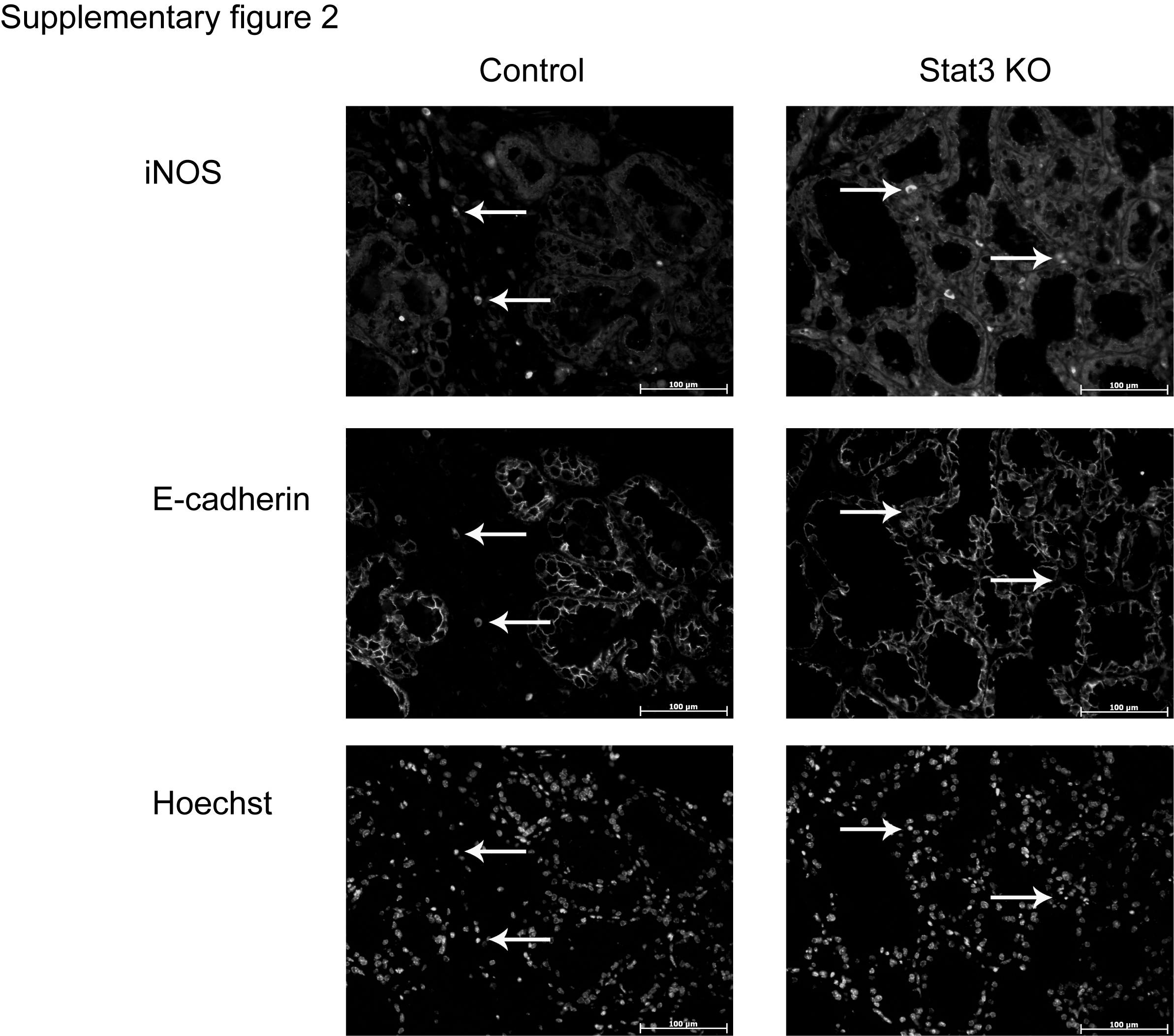

Supplement: Supplementary file 5 [file path0227-0106-SD2.tif]

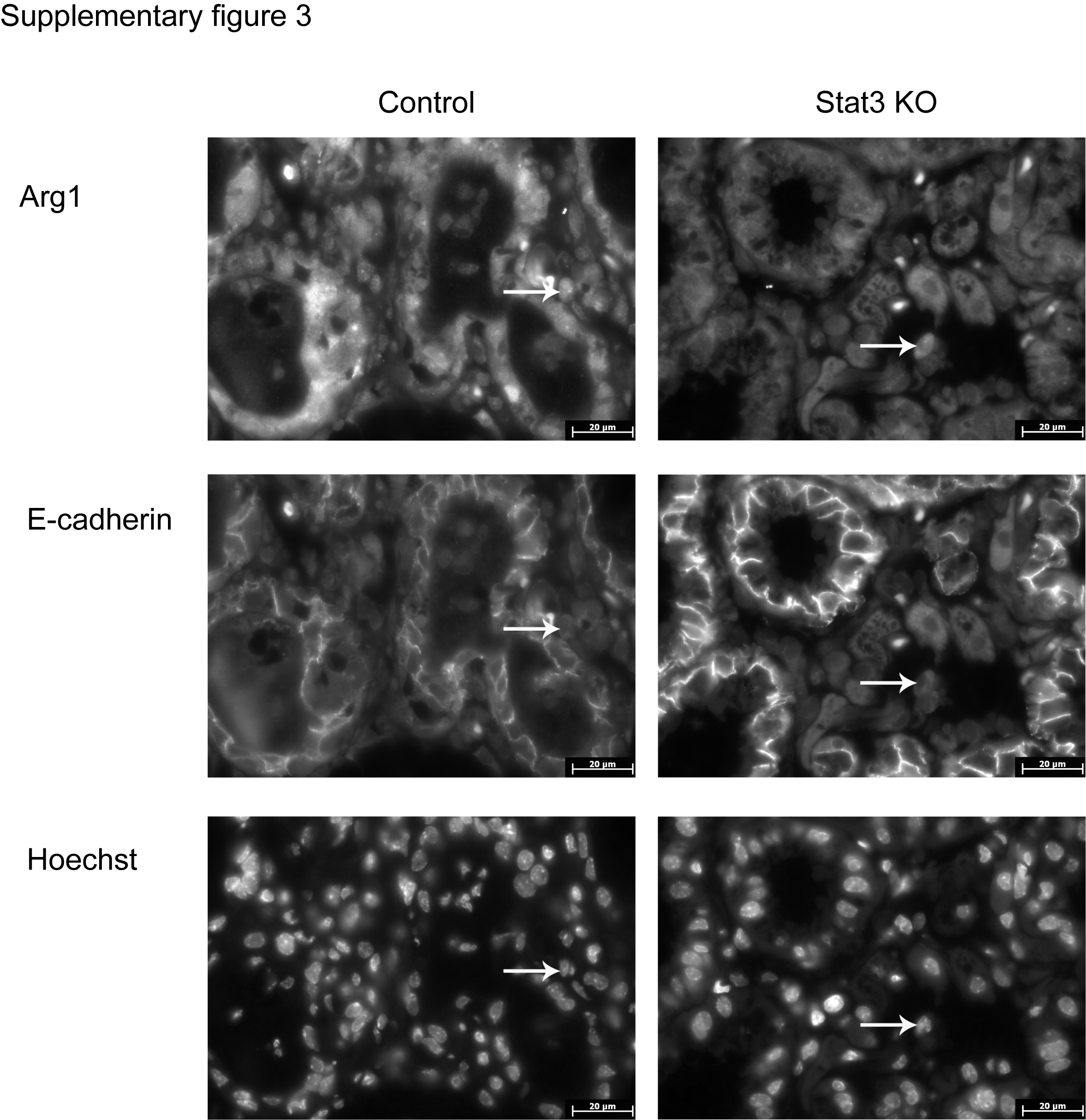

Supplement: Supplementary file 6 [file path0227-0106-SD3.tif]

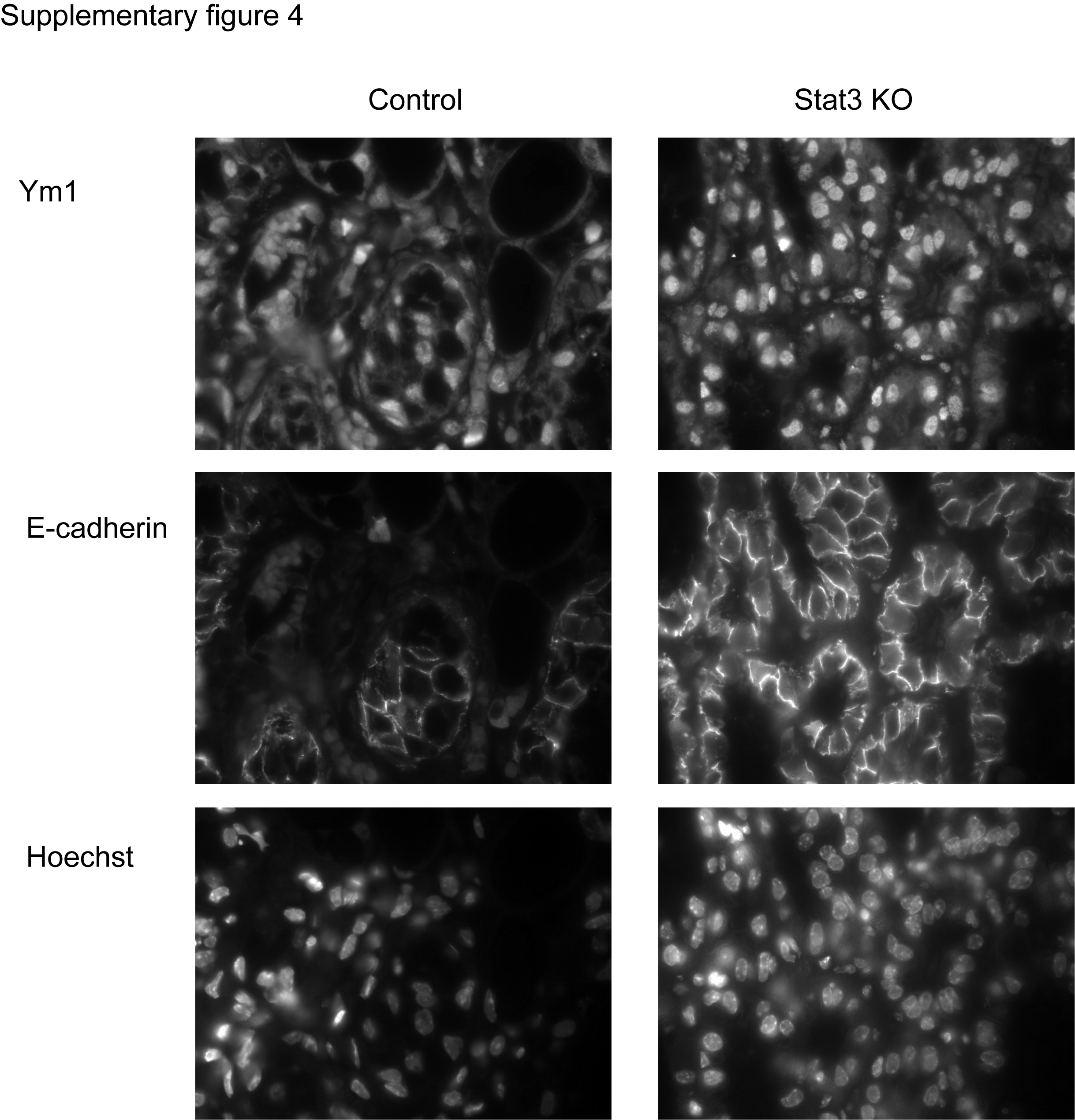

Supplement: Supplementary file 7 [file path0227-0106-SD4.tif]

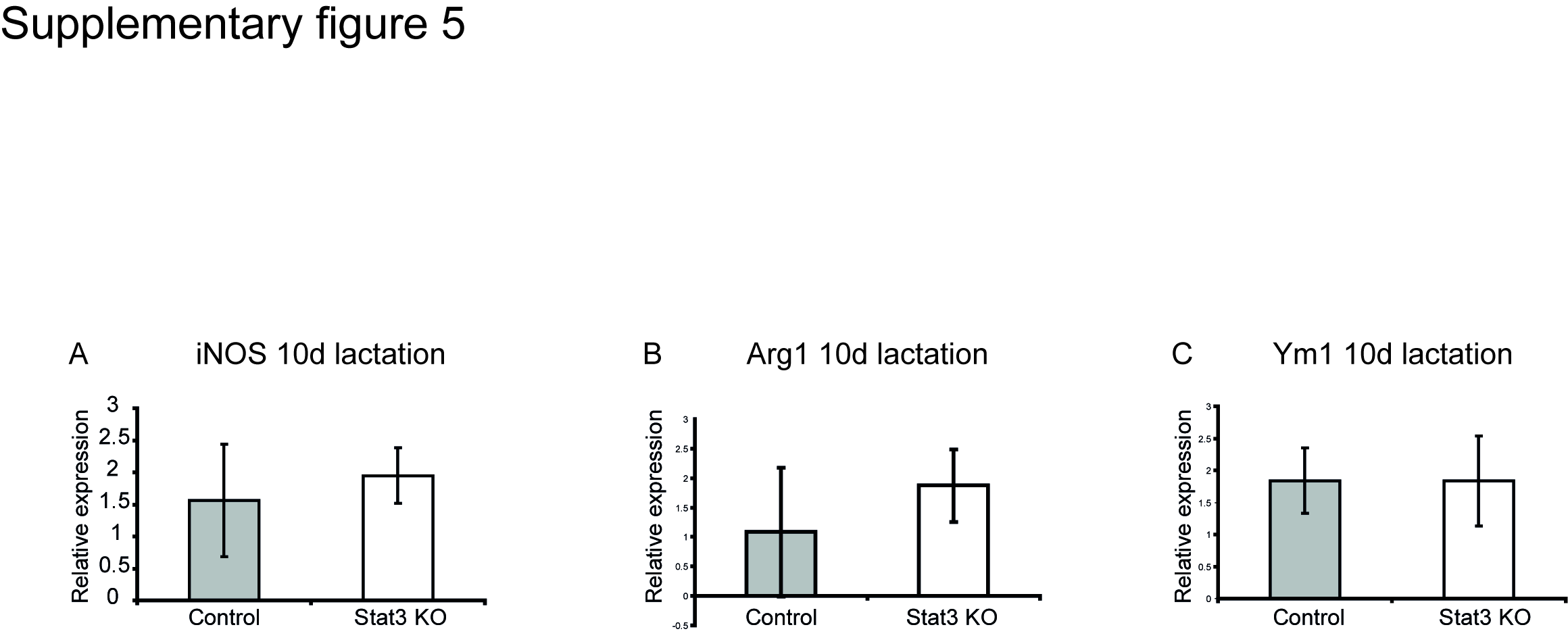

Supplement: Supplementary file 8 [file path0227-0106-SD5.tif]
