# Supplementary material for: Conditional deletion of Stat3 in mammary epithelium impairs the acute phase response and modulates immune cell numbers during post-lactational regression
Source: J Pathol. 2012 Jan 27;227(1):106–17. doi: 10.1002/path.3961 (PMC3477635; doi:10.1002/path.3961)
Supplement: Supplementary file 9 [file path0227-0106-SD9.doc]

**Table S1.** Primers used for quantitative RT–PCR analysis

| **Primer name** | **Sequence** |
| --- | --- |
| Cyclophilin | Fwd: CCTTGGGCCGCGTCTCCTT  Rev: CACCCTGGCACATGAATCCTG |
| CD14 | Fwd: ACAGGGGCTGCCAAATTGGTCG  Rev: AGCACACGCTCCATGGTCGGTA |
| Orosomucoid 1 (Orm1) | Fwd: CGAGTACAGGCAGGCAATTCA  Rev: ACCTATTGTTTGAGACTCCCGA |
| Orosomucoid 2 (Orm2) | Fwd: ATCTCTTCCAAGCCCTGGTGCCT  Rev: TTTAGGACAGCCGCACCAATGAA |
| Secretory leucocyte protease inhibitor (Slpi) | Fwd: GGCCTTTTACCTTTCACGGTG  Rev: TACGGCATTGTGGCTTCTCAA |
| Leucine rich glycoprotein 1 (Lrg1) | Fwd: TTGGCAGCATCAAGGAAGC  Rev: CAGATGGACAGTGTCGGCA |
| Chitinase 3-like 1 (Chi3L1) | Fwd: GTACAAGCTGGTCTGCTACTTC  Rev: ATGTGCTAAGCATGTTGTCGC |
| Matrix metalloproteinase 2 (MMP2) | Fwd: CAAGTTCCCCGGCGATGTC  Rev: TTCTGGTCAAGGTCACCTGTC |
| Matrix metalloproteinase 3 (MMP3) | Fwd: TGGAGATGCTCACTTTGACG  Rev: GCCTTGGCTGAGTGGTAGAG |
| Matrix metalloproteinase 9 (MMP9) | Fwd: GGACCCGAAGCGGACATTG  Rev: GAAGGGATACCCGTCTCCGT |
| Prekallikrein (Klkb1) | Fwd: TGGTCGCCAATGGGTACTG  Rev: ATATACGCCACACATCTGGATAGG |
| Inducible nitric oxide synthase (iNOS) | Fwd: GTTCTCAGCCCAACAATACAAGA  Rev: GTGGACGGGTCGATGTCAC |
| Arginase-1 (Arg1) | Fwd: CTCCAAGCCAAAGTCCTTAGAG  Rev: AGGAGCTGTCATTAGGGACATC |
| Ym1 | Fwd: AGAAGGGAGTTTCAAACCTGGT  Rev: GTCTTGCTCATGTGTGTAAGTGA |
| Interleukin 4 receptor*-α* (IL-4 receptor*-α*) | Fwd: GATTAAGAAGATATGGTGGGACCAGAT  Rev: CCTGAATGATGATGGCTGCTAAG |
